# TNF-α levels and presence of SNP-308G/A of TNF-α gene in temporomandibular disorder patients

**DOI:** 10.1590/2177-6709.27.1.e2220159.oar

**Published:** 2022-02-28

**Authors:** Camilla Porto CAMPELLO, Elker Lene Santos de LIMA, Renata Silva Melo FERNANDES, Mirza PORTO, Maria Tereza Cartaxo MUNIZ

**Affiliations:** 1 Universidade Federal Rural de Pernambuco, Programa de Pós-Graduação em Biotecnologia, Rede Nordeste de Biotecnologia (Recife/PE, Brazil).; 2 Universidade de Pernambuco, Faculdade de Ciências Médicas, Programa de Pós-Graduação em Ciências da Saúde (Recife/PE, Brazil).; 3 Universidade de Pernambuco, Hospital Universitário Oswaldo Cruz, Laboratório de Biologia Molecular-CEONHPE (Recife/PE, Brazil).; 4 Universidade Federal de Pernambuco, Departamento de Prótese e Cirurgia Buco-Facial (Recife/PE, Brazil).; 5 Universidade Católica de Pernambuco, Assessoria de Treinamento, Estágio, Pesquisa e Integração - ASTEPI (Recife/PE, Brazil).; 6 Universidade de Pernambuco, Instituto de Ciências Biológicas (Recife/PE, Brazil).

**Keywords:** Temporomandibular disorder, Interleukin, Polymorphism

## Abstract

**Introduction:**

Temporomandibular disorder (TMD) refers to a group of conditions that compromise the harmonious movement and function of the temporomandibular joint, masticatory muscles, and associated structures. The etiopathogenesis of TMD is multifactorial but not well-understood, with the role of genetic factors still being unclear.

**Objective:**

This review aims to summarize the results of studies that evaluated TNF-α levels and the -308G/A TNF-α polymorphism in TMD patients. This study emphasizes the importance of a more selective treatment involving TNF-α inhibitors that can potentially reduce inflammation and pain, and improve quality of life.

**Methods:**

The MEDLINE/PubMed database, Cochrane Library, and Web of Science database were searched for case-control studies published until September 2020 that compared levels of TNF-α or presence of its -308G/A polymorphism in TMD patients and healthy individuals.

**Results:**

Six case-control studies were identified with a total of 398 TMD patients, aged between 12 and 78 years. The control group consisted of 149 subjects, aged between 18 and 47 years. The occurrence of TMD was predominant in females. Majority of studies found high TNF-α levels in TMD patients, compared to the control group. One of these studies found a positive correlation between the GA genotype and the development of TMD.

**Conclusion:**

Majority of the TMD patients showed elevated TNF-α levels, and a possible explanation for this could be the presence of the -308G/A polymorphism.

## RESUMO

**Introdução:**

A disfunção temporomandibular (DTM) é definida como um grupo de alterações que comprometem a articulação temporomandibular, os músculos mastigatórios e as estruturas associadas. A etiopatogenia da DTM é multifatorial, e o papel dos fatores genéticos permanece obscuro.

**Objetivo:**

A presente revisão teve como objetivo descrever as contribuições de estudos que avaliaram os níveis de TNF-α e o polimorfismo -308 G/A em pacientes com DTM. Esse estudo enfatizou a importância de um tratamento mais completo envolvendo os inibidores do TNF-α que podem potencialmente reduzir a inflamação e a dor, contribuindo para melhorar a qualidade de vida do paciente.

**Métodos:**

As pesquisas foram realizadas nas bases de dados MEDLINE/PubMed, Cochrane Library e Web of Science, em busca de estudos de caso-controle publicados até setembro de 2020 que avaliassem os níveis de TNF-α e seu polimorfismo -308 G/A nos pacientes com DTM e em controles saudáveis.

**Resultados:**

Seis estudos de caso-controle foram identificados, com um total de 398 pacientes com DTM, e a idade variou de 12 a 78 anos. O grupo controle consistiu de 149 indivíduos e sua idade variou, aproximadamente, de 18 a 47 anos. O sexo feminino foi predominante. A maioria das pesquisas encontrou níveis elevados de TNF-α nos pacientes, em comparação com os controles. Um estudo encontrou uma associação positiva entre o genótipo GA e o desenvolvimento de DTM.

**Conclusão:**

A maioria dos pacientes com DTM demonstrou predisposição a uma maior produção de TNF-α, e isso poderia ser explicado pela presença do polimorfismo -308 G/A.

## INTRODUCTION

Temporomandibular disorder (TMD) refers to a group of conditions that compromise the harmonious movement and function of the temporomandibular joint (TMJ), masticatory muscles, and associated structures.^1^ Chronic TMD commonly occurs as orofacial pain, and is considered a public health problem.^2^ It occurs more frequently and severely in women than in men.^3^ Its prevalence in the Brazilian population is between 4% and 12%.^4^ The most common symptoms of TMD are limited mandibular movements, TMJ sounds (click and crepitus), headache, and pain.^5^

The etiopathogenesis of TMD is multifactorial and involves joint and muscle trauma, anatomical factors, psychosocial aspects, and sensitization of nociceptive pathways, but the role of genetic factors in the etiology of TMD remains unclear and needs to be investigated.^6^ As TMD is caused by multiple factors, several forms of treatment are available, such as occlusal splints, counseling, physical therapy,^7^ surgery, acupuncture, botulinum toxin injection, pharmacotherapy,^8^ orofacial myofunctional therapy, and low laser therapy.^9^ Pharmacotherapy involves anti-inflammatory agents, analgesics, muscle relaxants, and in certain situations, tricyclic antidepressants; however, in certain cases, these therapeutics are not successful and patients suffer with persistent pain.^8^

In recent decades, a considerable amount of research data has accumulated in the field of TMD, and therapeutic techniques have also improved remarkably. Despite all the new information and alternative therapies that have come to light,^10^ there is no effective therapy in some cases, and patients are forced to suffer prolonged debilitating pain and have a poor quality of life. Thus, it is crucial to investigate the relationship between the genetic profile and development of TMD^4^, because it can help develop new therapeutic methods. Therefore, it is essential to monitor interleukin concentrations and identify gene polymorphisms that can modify interleukins levels. 

Tumor necrosis factor alpha (TNF-α) is an important pro-inflammatory cytokine that contributes considerably to inflammation and immune response.^11^ TNF-α is mainly produced by macrophages, lymphocytes, and trophoblastic cells.^12^ The single nucleotide polymorphism (SNP) -308G/A TNF-α rs1800629 is characterized by the replacement of guanine (G) with adenine (A) in the promoter region of the gene, which leads to a greater production of this interleukin.^13^

Only a few studies have evaluated the presence of interleukins and genetic polymorphisms in TMD patients, but they have provided important results regarding the role of tumor necrosis factor. This has pointed to the possibility of the inclusion of this immune-inflammatory marker of immunity in the evaluation of TMD, as well as in the development of a more selective treatment based on monoclonal antibodies.^14^ This review aims to summarize the results of studies that evaluated TNF-α levels and the -308G/A TNF-α polymorphism in TMD patients. These studies lead us to reflect on the importance of a more selective treatment involving TNF-α inhibitors that can potentially reduce inflammation and pain, and improve quality of life.

## MATERIAL AND METHODS

### ELIGIBILITY CRITERIA

Only those case-control studies that evaluated levels of TNF-α or its -308G/A polymorphism in TMD patients were included. Studies focusing on other polymorphisms and/or interleukins, and studies that did not draw comparison to a control group were excluded.

### INFORMATION SOURCES AND SEARCH STRATEGY

The MEDLINE/PubMed database, Cochrane Library, and Web of Science database were searched for case-control studies published until September 2020 that compared TNF-α concentrations and presence of its -308G/A polymorphism in TMD patients and healthy controls. The following search terms were used for this purpose: “Temporomandibular Joint disorders Interleukin”, “Temporomandibular Joint Disorders Polymorphism, “Temporomandibular Dysfunction Interleukin,” and “Temporomandibular Dysfunction Polymorphism.”

Furthermore, OpenGrey (www.opengrey.eu) was used for gray literature research. The studies were selected on the basis of their titles and summaries. 

### DATA COLLECTION PROCESS

The following variables were collected: author, type of study, number of patients, number of healthy individuals, gender, mean age, TNF-α levels, and the presence of the -308G/A polymorphism of this interleukin. 

## RESULTS

All reports considered in this review are case-control studies that measured TNF-α concentrations or detected the presence of -308G/A polymorphism in TMD patients. Six studies were identified, with a total of 398 TMD patients, aged between 12 and 78 years, whereas the control group consisted of 149 subjects, aged between 18 and 47 years. Details of the six studies included in this review are described in Table 1. 

**Table 1:** Profile of TMD patients and healthy controls.

	Study, year	(n)		Gender		Age (years)	
		Patients	Controls	Patients	Controls	Patients	Controls
TMD TNF-α levels	Takahashi et al.^15^, 1998	51	6	46 females	0 females	17-78	27-39
		62 TMJ	10 TMJ	5 males	6 males		
	Kaneyama et al.^16^, 2002	117	7	123 females	0 females	12-74	27-35
		121 TMJ	9 TMJ	14 males	7 males		
	Lee et al.^17^, 2010	24	5	Not informed	Not informed	Not informed	Not informed
		24 TMJ	5 TMJ				
	Park, Chung^18^, 2016	40	20	40 females	20 females	32.9 ± 13.3	32.9 ± 13.3
				0 male	0 males		
	Louca Jounger et al.^19^, 2017	20	20	20 females	20 females	Above 18	Above 18
				0 male	0 male		
TMD SNP	Furquim et al.^20^, 2016	152	91	136 females	82 females	36.07 ± 11.00	34.68 ± 11.47
				14 males	9 males		

n = number, TMJ = temporomandibular joint.

Most patients in these studies were diagnosed according to the Research Diagnostic Criteria for Temporomandibular Disorders (RDC/TMD).^21^ Five studies evaluated TNF-α levels in TMD patients,^15^ ^-^ ^19^ which were expressed as mean concentration ± SD (Table 2), and one study detected the presence of the -308G/A polymorphism of this interleukin^20^ (Table 3). Most of these studies found higher TNF-α levels in the TMD patients group than in the healthy individuals.^16^ ^,^ ^18^ ^-^ ^19^

**Table 2:** TNF-α levels in TMD patients and control group.

Studies	Diagnostic criteria	Diagnostic (n)	Sample type	Patients	Controls	*p* **-value**
Takahashi et al.^15^	Reciprocal click in the joint, joint pain and short-term intermittent block for DD with click. TMJ block, impairment of joint mobility, joint pain and history of intermittent clicks and blocks for intermittent disc displacement. Impaired joint mobility, joint pain and bone degenerative changes in the joint surface observed in tomography and magnetic resonance for TMJ-OA	DD with click (8) DD with locking (25) TMJ-OA (18)	Synovial fluid	Detected in 5 joints	Not detected	>0.05
Kaneyama et al.^16^	The same of Takahashi et al.^15^	DD with click (8) DD with locking (54) TMJ-OA (59)	Synovial fluid	0.03 pg / mL DD with click; 0.17 pg / mL DD with locking; 0.17 pg / mL TMJ-OA	Not detected	<0.05
Lee et al.^17^	Pain, mouth opening limitation and clicking	TMD acute pain (14) TMD chronic pain (10)	Synovial fluid	0.39± 0.05 pg/mL	0.36 ± 0.03pg/L	0.05
*Park, Chung^18^	RDC/TMD^21^	Greater disability (20) Lesser disability (20)	Plasma	4.55 pg / mL Greater disability; 1.86 pg / mL Lesser disability	0.11 pg / mL	≤ 0.001
^#^Louca Jounger et al.^19^	RDC/TMD^21^	TMD Myalgia (20)	Masseter muscle	10 pg / mL After repetitive dental tightening	3.5 pg / mL After repetitive dental tightening	0.042

n = number of patients, P = X^2^, * Kruskal-Wallis test , # U test, DD = disc displacement, TMJ-OA = TMJ osteoarthritis, RDC/TMD = Research Diagnostic Criteria for Temporomandibular Disorders.

**Table 3:** Genotype distribution of the -308G/A SNP in patients and controls.

Study	Diagnostic criteria	Diagnostic (n)	Sample type	Patients genotype	Controls genotype	P	OR genotype GA	P^O^
Furquim et al.^20^	RDC/TMD^21^	TMD (152)	Synovial fluid	GG - GA - AA 111 - 32 - 9	GG - GA - AA 79 - 8 - 4	<0.05	2.873	0.0116

P= X^2^, OR=Odds ratio, P^Ο^= P (OR).

## DISCUSSION

This literature review included six articles that analyzed TNF-α levels or the -308G/A polymorphism in TMD patients, and compared their results with those of a control group. A total of 398 patients were evaluated, consisting of 346 females and 28 males, aged between 12 and 78 years. Age and gender of 24 of these patients were not specified. The prevalence of this disorder in females and in similar age groups has also been reported in other studies.^4^ ^,^ ^9^ ^,^ ^22^ ^,^ ^23^

Of the six studies analyzed, five evaluated TNF-α concentrations and one investigated the presence of the -308G/A polymorphism, which is a G to A mutation at the -308 position in the promoter region, and is associated with higher levels of TNF-α.^13^ Despite the fact that these studies are difficult to compare because different fluids were used to measure the concentration of this interleukin, all lead us to reflect on the possibility of developing a more targeted treatment. Two articles^16^ ^,^ ^18^ found a significant difference in TNF-α levels of patients compared to controls, which indicated that anti-TNF drugs may be prescribed for these patients. The first investigation^16^ illustrated that TNF-α concentration levels in patients with TMD disc displacement with locking (54) and TMJ-osteoarthritis (59) were greater than those in patients with TMD disc displacement with click (8). Nevertheless, there is a significant difference between the sample sizes of the TMD subgroups. Park and Chung^18^ found elevated TNF-α levels in a group with greater TMD disability (20) in comparison to a group with lesser disability (20), indicating that this interleukin level is higher in patients with greater disability.

Furquim et al.^20^ identified that the GA genotype of the -308G/A polymorphism was significantly greater in patients than in controls. These findings could explain the elevated concentrations of this interleukin in patients evaluated by Kaneyama et al.,^18^ because the GA genotype is associated with an intermediate production of TNF-α, and the GG genotype is related to a low production of this interleukin.^24^ Further experimental studies evaluating this polymorphism and TNF-α levels are required to confirm these results.

Louca Jounger et al.^19^ evaluated TNF-α concentrations in masseter muscle and detected an increased concentration of this interleukin in patients compared to controls when measured after 160 min of evaluation and repetitive dental tightening. This data illustrates a patient’s predisposition to a greater production of TNF-α, and a possible reason for this could be the presence of the -308G/A polymorphism in TMD patients.

Similarly, Lee et al.^17^ found higher TNF-α levels in patients than in controls; however, there was no statistical difference between these groups (*p*= 0.05). This study had fewer patients (24) compared to other studies that measured TNF-α levels in synovial fluid. The sample size of the patients may have influenced the results. Additionally, this study included cases with acute and chronic pain; however, there was no statistically significant difference in TNF-α levels between these groups.

Takahashi et al.^15^ did not detect any difference in TNF-α levels between patients and healthy subjects. In this study, the patients had been treated for three months, which could have reduced inflammation and consequently the levels of interleukins. Medications were suspended at least two weeks prior to the synovial fluid collection; however, the treatment may have already helped to control the inflammation. In addition, patients were using an occlusal splint and undergoing physiotherapy, which can also decrease inflammation. There is a possibility that these treatments could have influenced the results. Furthermore, the control group only comprised men.

A recent study in transgenic mice observed that high concentrations of TNF-α cause catabolic changes that considerably affect the TMJ, causing irregular bone erosion and loss of cartilage.^25^ The SNP -308G/A in the promoter region^13^ is associated with higher levels of TNF-α.^24^ The A allele and AA genotype of the SNP -308G/A are associated with an increase in TNF-α production.^13^ ^,^ ^24^ Five studies described in this review analyzed TNF-α levels, and one evaluated the presence of the SNP -308G/A TNF-α. It is paramount to focus research efforts towards comparing TNF-α levels and its -308G/A polymorphism in the same patients.

Identifying inflammatory cytokines involved in TMD can help establish an innovative therapy or the development of new drugs.^26^ Genetic polymorphisms can provide relevant information about an individual’s health status, the risk of development of TMD or its severity, and specific treatment options.^27^ TNF-α inhibitors reduce the risk of joint damage, improve physical function, and consequently, the quality of life of patients with rheumatoid arthritis,^28^ an autoimmune disease that causes chronic pain and joint pain, including TMJ.^29^ New investigations on these medications could also have significant therapeutic value in individuals with TMD.

Further research is required not only to measure TNF-α concentrations and identify the -308G/A TNF-α polymorphism, but also to detect other interleukins and polymorphisms that could be inflammatory markers in TMD cases. It is essential to detect TMD risk factors that can predispose subjects to develop TMD or aggravate this disorder. It is necessary to develop a more specific treatment to reduce or eliminate pain and promote a better quality of life for these patients.

It is also important to conduct tests that assess the psychological status and somatosensory profiles of TMD patients, because these assessments can show different effects in patients’ pain profile; consequently, they could contribute to understanding the pain mechanism, its maintenance, and treatment guidelines.^23^

An investigation demonstrated that centrally mediated myalgia and TMD with disc displacement had higher pain intensity than masticatory myofascial pain, local myalgia, capsulitis/synovitis, and continuous neuropathic pain, which could be explained by genetic, psychological, social, or behavioral aspects.^30^ Elevated levels of TNF-α and its -308G/A polymorphism, which is associated with higher concentrations of this interleukin,^13^ ^,^ ^24^ could be present in patients with greater pain intensity.

The limitations of the studies analyzed in this review were the small sample sizes considered in some studies, different diagnostic criteria, and the fact that while some studies involved both women and men, some control groups consisted only of men. It is essential to follow the same diagnostic criteria, taxonomy, and nomenclature, because research questions and findings can be standardized; consequently, clinicians can better diagnose and monitor their patients.^5^ Few studies have evaluated the TNF-α profile in patients with TMD. Our suggestion for similar studies in future is to work with larger sample sizes that have also women constituting the control group, because TMD is more severe in women.^3^ In addition, these studies analyzed interleukin levels in different fluid samples, like synovial fluid and blood, which makes it difficult to compare the articles. We suggest that TNF-α levels and the analyzed polymorphism can be used as inflammatory markers for developing a more targeted treatment protocol.

## CONCLUSION

Most TMD patients showed a predisposition to a greater production of TNF-α, which could be explained by the presence of the -308G/A polymorphism. Further investigations are required to confirm the results of this review and to analyze the role of other proinflammatory and anti-inflammatory interleukins and their polymorphisms in TMD.
